# Single-nucleotide polymorphisms link gout with health-related lifestyle factors in Korean cohorts

**DOI:** 10.1371/journal.pone.0295038

**Published:** 2023-12-07

**Authors:** Hye Kyung Jeon, Hae Young Yoo

**Affiliations:** 1 Department of Nursing, Ansan University, Gyeonggi-do, Korea; 2 Department of Nursing, Chung-Ang University, Seoul, Korea; Boston University Chobanian & Avedisian School of Medicine, UNITED STATES

## Abstract

Gout—a very painful inflammatory arthritis caused by the deposition of monosodium urate crystals in the joints—is influenced by several factors. We identified the association of single- nucleotide polymorphisms (SNPs) that link gout with health-related lifestyle factors using genomic data from the Korean Genome and Epidemiology Study. We conducted a genome-wide association study (GWAS) on 18,927 samples of 438 Korean patients with gout and 18,489 controls for the discovery stage. For the replication stage, another batch containing samples of 326 patients with gout and 2,737 controls were analyzed. Lastly, a meta-analysis was performed using these two cohorts. We analyzed the effects of health-related lifestyle factors, including eating habits, physical activity, drinking behavior, and smoking behavior, on gout. After identifying the association between GWAS-derived SNPs and health-related lifestyle factors, we confirmed the interaction between the polygenic risk score (PRS) and health-related lifestyle factors. We identified 15 SNPs related to gout, among which rs1481012 of *ABCG2* located on chromosome 4 has been newly discovered (*P* = 2.46e^-11^). On examining the interaction between SNPs and health-related lifestyles, rs3109823—located in *ABCG2*—was found to be associated with smoking status. In addition, rs11936395—located in *SLC2A9*—was significantly associated with the average momentum of exercise per session, whereas rs11066325 located in *PTPN11*, showed a significant association with the number of exercise sessions per week, smoking status, drinking status, and amount of soju drink per session. rs9421589—located in *FAM35A*—was significantly associated with the duration of smoking. In addition, we verified that the association between PRS and duration of smoking affects gout. Thus, in this study, we identified novel SNPs that link gout with health-related lifestyle factors in the Korean population.

## Introduction

Gout is a very painful inflammatory arthritis caused by the deposition of monosodium urate crystals in the joints due to abnormal purine metabolism and underexcretion of serum urate, causing inflammation and pain in the joints and the surrounding tissues [1]. It is influenced by a combination of genetic variations and environmental factors, and genetic factors being the key reason for renal excretion of serum urate. The change in serum urate concentration can be primarily (40–80%) attributed to genetic factors [2]. One of the genetic factors related to uric acid excretion by the kidneys is single nucleotide polymorphisms (SNPs). A SNP is a germline substitution of a single nucleotide occurring at the same location in a gene for different individuals. These genetic mutations have a varied prevalence depending on race and group [3].

With the advancement of genome-wide association study (GWAS), several genes associated with gout have been reported [4]. According to previous GWAS, the representative genes related to gout include *ABCG2* (encodes the strongest renal apical urate secretion transporter), *SLC2A9* (encodes renal basolateral GLUT9, the only urate reabsorption transporter that transports urate from proximal tubule epithelial cells to blood stream), *SLC17A1* (encodes NPT1, an apical renal urate secretion transporter), *SLC17A3* (encodes NPT4, an apical renal urate secretion transporter), *SLC22A11* (encodes OAT4, an apical renal urate reabsorption transporter), *SLC22A12* (encodes URAT1, the strongest apical renal urate/nicotinate exchanging reabsorption transporter), and *PKD2* (encodes polycystin-2, an integral membrane protein having characteristics of a calcium-permeant cation channel) [4–10]. Effects of the interaction between *PKD2* and *ABCG2* on gout has been studied in Chinese and Japanese male populations, respectively [5]. In addition, the genetic association between gout and rs2230054 of *CXCR2* in Chinese Han men has been recently studied [8]. In Korea, the association between electric genes and the interaction between genes in Korean gout was investigated through the Korean Association Resource (KARE) cohort [11]. Sull et al. [12] also investigated the impact of *SLC2A9* mutations on serum urate levels in Koreans. Additionally, a significant association between rs2231142 or rs2054576 of *ABCG2* and Korean gout was revealed [6, 13]. Consequently, prior studies on gout have had varied results based on race or study group.

In Korea, the number of patients with gout is increasing rapidly, and it is prevalent not only among those in their 50s and 60s but also among those in their 20s and 30s owing to the increase in meat intake and sedentary lifestyles, which leads to obesity [14]. The average serum urate concentration of the entire population is increasing in Korea, attributed to multifaceted factors such as lifestyle and dietary habits [15].

Epigenetics asserts that differences in the environment can modify gene expression. It also shows that expression of genes varies depending on the environment, diet, and behavioral habits [16]. Moreover, the gene–environment correlation does not exist independently, and changes in one affect the characteristics of the other through a feedback loop [17, 18]. Therefore, it is necessary to promote good health by enhancing environmental factors owing to the role the interaction of genetic and environmental factors play in human diseases. In addition, although gout is a disease affected by both genetic variations and environmental factors, previous studies on genetic variations related to gout in Korean patients with gout focused on only the genetic aspect without considering environmental factors. Therefore, detailed research considering both environmental and genetic factors in Korean gout is urgently needed.

In this study, we conducted a GWAS analysis on patients with gout in Korea based on the Health Examinees study (HEXA) cohort and KARE cohort of the Korean Genome and Epidemiology Study (KoGES). Furthermore, the polygenic risk score (PRS) was calculated as a weighted sum of the risk alleles of the SNPs to assess the risk level of the SNPs identified in the GWAS analysis [19]. PRS has been used extensively to predict the genetic predisposition toward diseases, since it was established that the genetic risk for schizophrenia is a predictor of bipolar disorder [20]. Ultimately, this study seeks to identify the SNPs related to gout in Koreans, analyze their interactions with health-related lifestyle factors, and determine whether these interactions affect gout. In addition, this study calculated the PRS to analyze the genetic risk of gout in Koreans and assessed the interaction between PRS and health-related lifestyle factors.

## Material & methods

### Data collection

We used the epidemiologic and genomic data from the first HEXA-based survey conducted from 2004 to 2013 from the KoGES, as well as the genomic data from the first KARE-based survey conducted from 2001 to 2002, from the KoGES, conducted by the Korea Centers for Disease Control and Prevention. The Korean Biobank Array (also called the K-Chip or KoreanChip) was used to process the HEXA and KARE genomic data.

### Study participants

This study included data from subjects in the age group of 40 to 69 years, recruited by the KoGES. Among the 173,208 people who were included in the HEXA-based survey, the epidemiologic and genomic data of 58,700 people, including their genetic information, were used. Similarly, the genomic data of 5,493 people out of the 10,031 people studied in the KARE-based study were used.

In the discovery stage, a total of 18,927 subjects were analyzed, including the 438 self-reported subjects with gout in the HEXA cohort. It was assumed that gout is diagnosed by rheumatologists according to the American College of Rheumatology diagnostic criteria [21].

Subjects who met any of the following criteria were excluded: “No response” (n = 7,624) and “Other diseases” (n = 32,149) such as chronic, metabolic, cardiovascular, cerebral, and respiratory diseases, detected through disease history investigation. In the replication stage, a total of 3063 subjects were analyzed, including the 326 subjects with gout in the KARE cohort (S1 Fig). This study was conducted according to the guidelines of the Declaration of Helsinki and approved by the institutional review boards of Chung-Ang University (approval no.: 1041078-202005-HRBR-137-01) and Korean center for Disease Control and Prevention (KBN-2020-080). All participants provided their written informed consent voluntarily.

### General and clinical characteristics

The data in this study were investigated using self-reported questionnaires and clinical examinations. The questionnaire was developed by including items from a semi-quantitative food frequency questionnaire, socio-demographic status, lifestyle (i.e., diet, smoking, drinking, and physical activity), and disease history for the KoGES [22].

Parameters like gender, age, marital status, occupation, education, monthly household income, medical history (hypertension/diabetes), age during gout diagnosis, height, weight, body mass index (BMI), systolic blood pressure, diastolic blood pressure, pulse, fasting blood sugar, blood urea nitrogen, creatinine, uric acid, total cholesterol (TC), low density lipoprotein cholesterol (LDL-C), high density lipoprotein cholesterol (HDL-C), triglycerides (TG), aspartate transaminase (AST), alanine transaminase (ALT), and high sensitivity C-reactive protein (hs-CRP) were analyzed. Bio-specimens included fasting blood samples that were collected in a serum separator tube and two ethylenediaminetetraacetic acid (EDTA) tubes, and a 10 ml midstream urine sample. For long-term storage, both serum and plasma were prepared and aliquoted in 6–10 vials (300–500 μl per vial), and 80–100 μg samples of blood DNA were also prepared [22].

### Health-related lifestyle factors

Health-related lifestyle factors are environmental factors such as eating habits, physical activity, drinking behavior, and smoking behavior. Eating habits included the number of regular meals per day and the average frequency of food intake over the past year. Physical activity included the exercise status, number of exercise sessions per week, and average momentum of exercise per session. Drinking behavior included drinking status, duration of drinking, average frequency of alcoholic beverages (soju/beer) consumed over the past year, and quantity of alcohol consumed (average of the amount soju/beer drink per session: one bottle of soju = 6.5 cups, 50 cc per cup/one bottle of beer = 2.5 cups, 220 cc per cup). Smoking behavior included smoking status, duration of smoking, and number of cigarettes smoked per day.

### Genetic data refinement and extraction

Data purification and SNP detection along with the analysis of the interaction between SNPs and health-related lifestyle factors were performed using the Plink (v.1.9, http://pngu.mgh.harvard.edu/purecell/plink/) genome analysis program. Functional Mapping and Annotation of Genome-Wide Association Studies was used to interpret the genomic data results and express them through Manhattan plot and Q–Q plot [23]. The SNPs for meta-analysis results were visualized using PhenoGram [24]. Locuszoom version 1.1 (http://csg.sph.umich.edu/locuszoom) was utilized to confirm the association between SNPs of *ABCG2* on chromosome 4 and to create regional plots [25].

Genomic DNA samples were genotyped using the Korea Biobank Array (K-chip). The K-chip is a new array, a Korean-customized dielectric chip developed by the Korea National Institute of Health in 2015. The K-chip consists of approximately 830,000 markers, including more than 247,000 rare-frequency and functional variants estimated through the sequencing data of more than 2,500 Koreans [26]. In this study, genotyping was conducted to determine the genotypes of 64,193 people and investigate the genotypes for 6,176,658 SNPs. In addition, SNP quality control (QC) was performed to purify genomic data to analyze 18,927 of the 58,700 subjects from the HEXA study included in the K-chip. Finally, 5,442,390 SNPs were used for actual analysis after QC by applying minor frequency (MAF) <0.05, SNP call rate <0.95, and Hardy–Weinberg equilibrium (HWE) P-value < 1E-6.

### Discovery stage: HEXA

In this study, the genomic data from the HEXA study included in the K-chip were analyzed to perform GWAS. Similar to that in a previous study [11], age, gender, and BMI were used as covariates and GWAS was performed using logistic regression analysis. We analyzed 18,927 out of 58,700 subjects in the HEXA study, and 15 lead SNPs significant for gout were detected. Lastly, the identified SNPs (n = 15) were assigned points (0, 1, or 2) based on the number of risk alleles present; the total score was equivalent to PRS.

### Replication stage: KARE

A replication study was performed on 3,063 subjects from the KARE cohort, with 326 subjects in the gout group and 2,737 subjects in the control group, to examine whether the 15 SNPs derived from the HEXA study were reproduced in this cohort. As with the discovery stage, age, gender, and BMI were adjusted as covariates. A meta-analysis was performed after the replication stage. Lastly, the PRS calculation method was the same as that in the discovery stage.

### Statistical analysis

Meta-analysis was performed by logistic regression analysis using PLINK by combining the discovery stage and replication stage of GWAS. The combined subject was composed of 764 cases and 21,226 controls.

Health-related lifestyle factors with SNPs of HEXA were analyzed by univariate logistic regression using PLINK. Binary logistic regression was performed with health-related lifestyle factors and PRS as independent variables, and the presence or absence of gout as a dependent variable.

## Results

### General and clinical characteristics

Based on gender, the gout subjects were categorized as male (369, 84.2%) and female (69, 15.8%), with relatively higher male subjects and significant differences between gout and healthy control group (χ^2^ = 559.32, *P*<0.001). The age of gout diagnosis was in the range of 50.31±9.02 years in the gout group. The serum urate concentration was 6.67±1.84 mg/dl in the gout group and 4.51±1.18 mg/dl in the control group, with significantly higher serum urate levels in the gout group (t = -24.46, *P*<0.001). The TC and LDL-C levels were significantly higher in the control group than in the gout group. HDL-C level was significantly lower in the gout group than in the control group (t = 14.33, *P*<0.001), whereas the TG, AST, ALT, and hs-CRP levels were significantly higher in the gout group (S1 Table).

### GWAS

**Fig 1:**
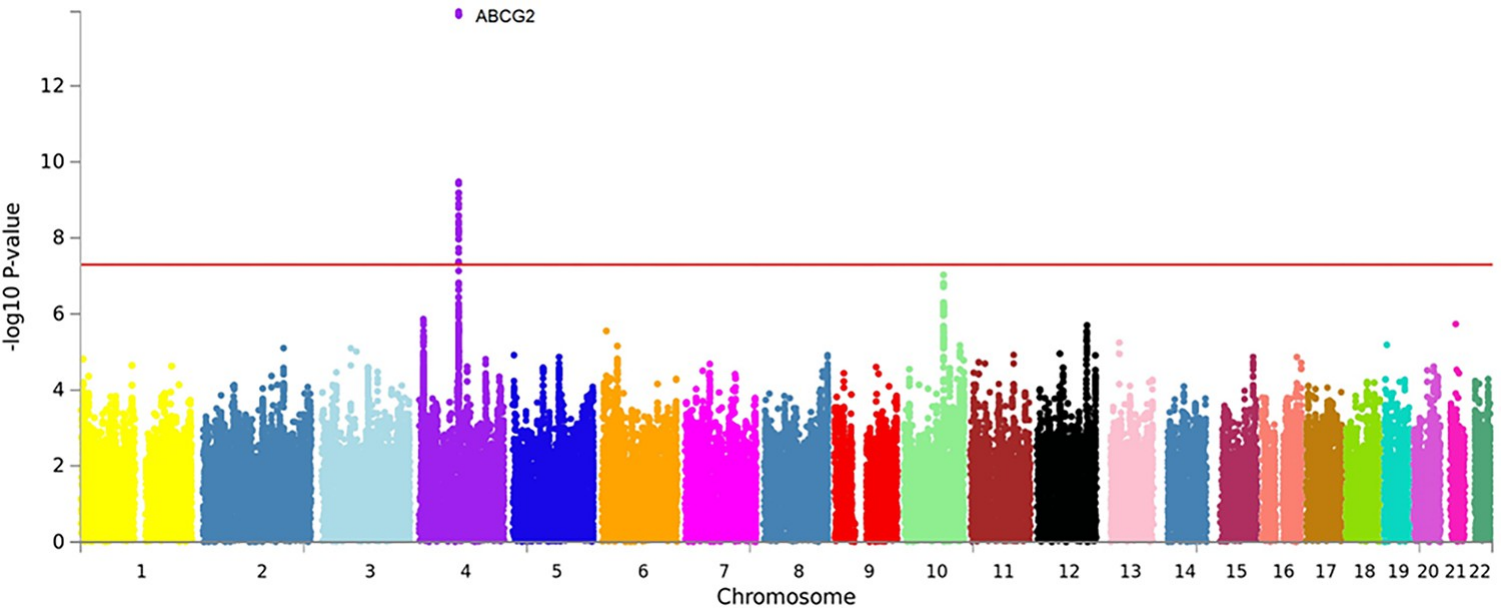
Manhattan plot of GWAS on HEXA. Manhattan plot showing the results of the GWAS analysis of patients with gout in the HEXA study. The X-axis of the plot shows the genomic location and the Y-axis shows the level of association and each dot represents an SNP. Significant SNPs associated with gout were identified in the genetic region associated with gout by the appearance of SNPs with a p-value of 1e^-5^ or less rising upward. The red line shows critical values for genome-wide statistical significance (*P*<1e^-5^).

**Fig 2:**
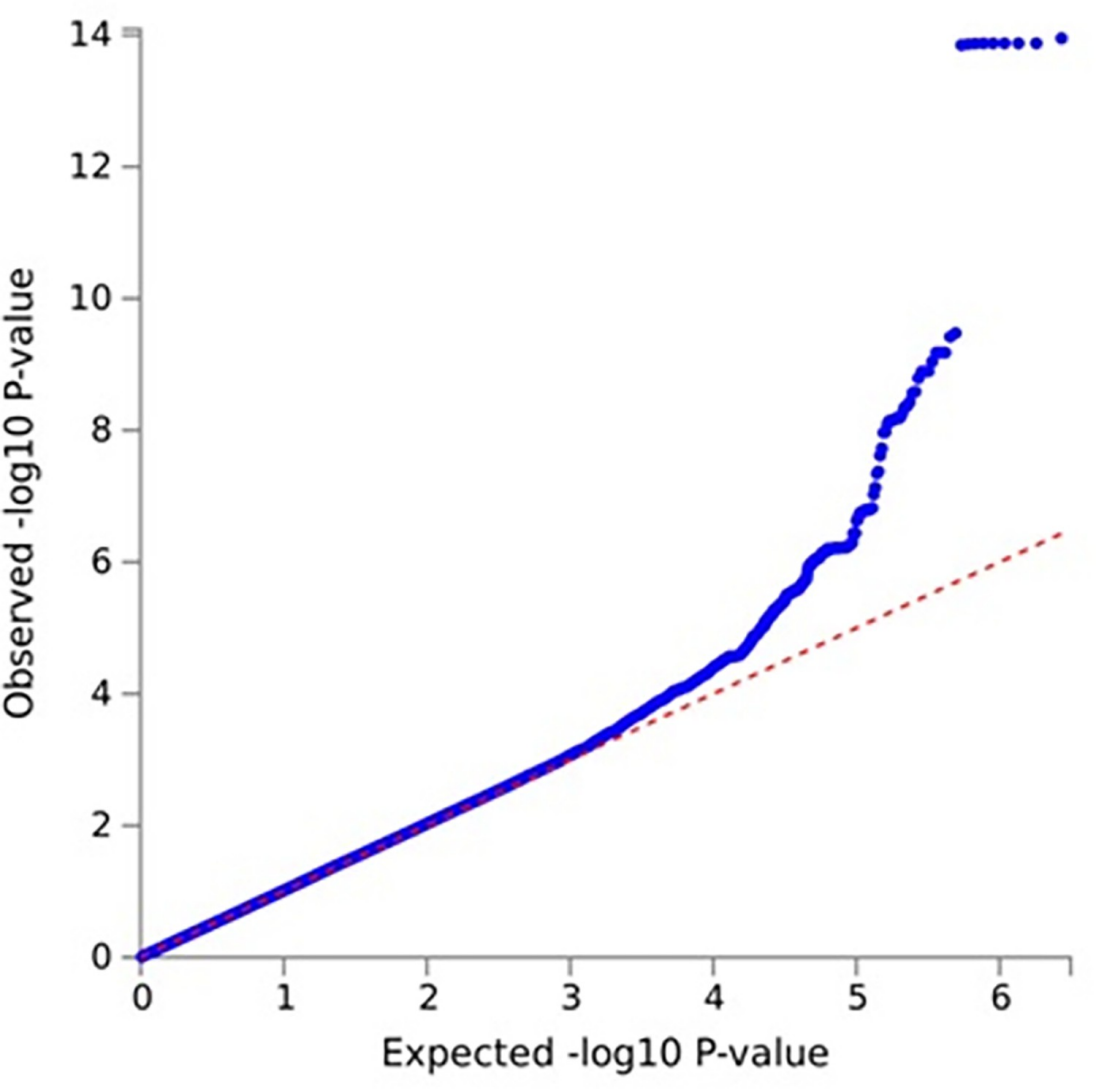
Q–Q plot of GWAS on HEXA. Q–Q plot showing the results of the GWAS analysis of patients with gout in the HEXA study. The blue dots representing SNPs coincide with the red oblique line below. They then deviate from the oblique line around the middle. SNPs that do not coincide with the oblique line are considered to be associated with gout.

**Discovery stage.** GWAS analysis, which was conducted to identify the genetic variants associated with gout in all the subjects in the discovery stage, revealed the presence of 15 SNPs. The most significant SNP was rs1481012 (*P* = 1.12e^-14^) located in *ABCG2*, based on the GWAS results. The second most significant SNP was rs11936395 (*P* = 1.37e^-06^) located in *SLC2A9* and rs200888518 (*P* = 1.84e^-06^) located in *APP*. The rest of the SNPs in order of their significance are as follows: rs11066325 (*P* = 2.00e^-06^) of *PTPN11*, rs3798728 (*P* = 2.80e^-06^) of *RP3-510L9*.*1*, rs9532070 (*P* = 5.71e^-06^) of *RP11-14O22*.*1*, rs3109823 (*P* = 6.02e^-06^) of *ABCG2*, rs339405 (*P* = 6.50e^-06^) of *TRIP10*, rs28674878 (*P* = 6.72e^-06^) of *CHST15*, rs56205418 (*P* = 6.98e^-06^) of *CLPS*, rs17794144 (*P* = 7.90e^-06^) of *CWC22*, rs17013965 (*P* = 7.98e^-06^) of *PPM1*, rs59517147 (*P* = 8.00e^-06^) of *KADAMTS9*, rs9421589 (*P* = 9.42e^-08^) of *FAM35A*, and rs146386352 (*P* = 9.76e^-06^) of *ROBO2* (S2 Table) (Figs 1 and 2).

#### Replication stage

In this stage, it was confirmed whether the 15 SNPs derived from the HEXA study in the discovery stage were reproduced in the KARE study as well (S3 Table).

#### Meta-analysis results

**Fig 3:**
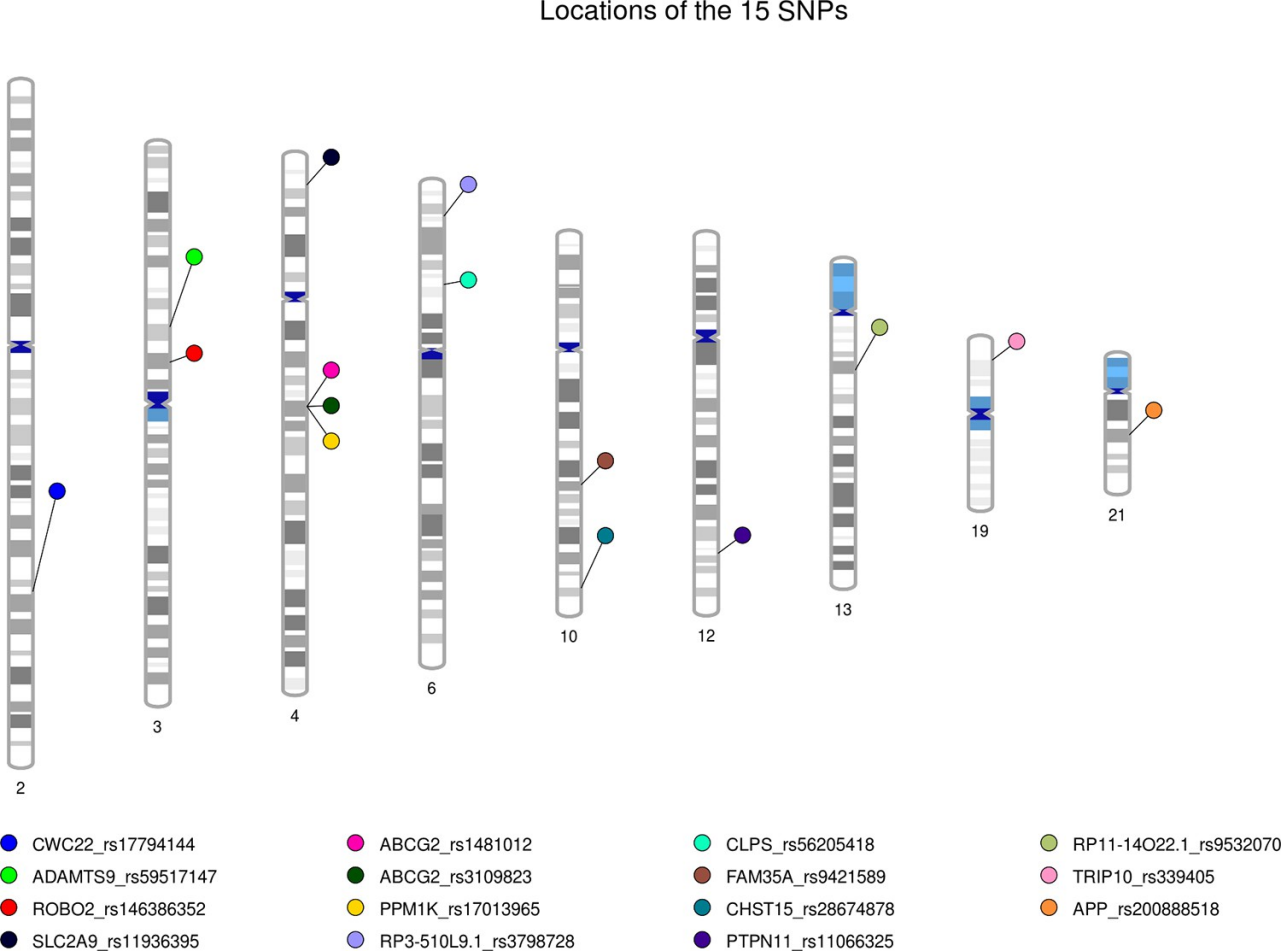
PhenoGram plot of gout. PhenoGram plot of the SNPs associated with gout. The long bars represent the 9 out of 22 chromosomes in which gout-related SNPs were found. The position of the SNP on each chromosome is indicated by a line and the line is connected to colored circles; Each different colored circles represent gout-related SNPs.

**Fig 4:**
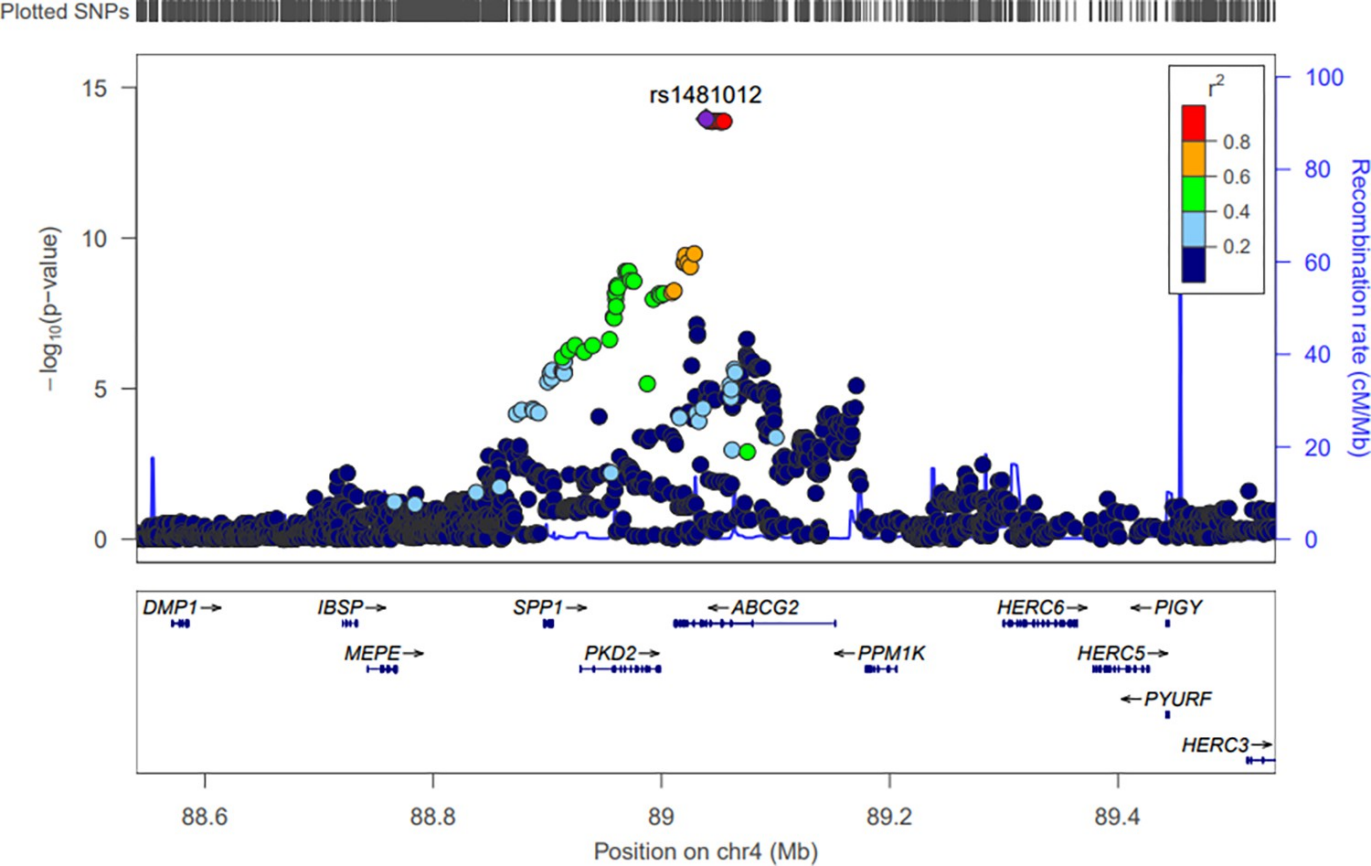
Regional plot of SNPs of *ABCG2* on chromosome 4. Regional plots were based on hg 19 version ASN (Asian population). A high r^2^ SNP was observed around rs1481012, which has the highest statistical significance. The purple diamond is an SNP that is the standard for regional plots, and rs1481012 showed the highest level of significance as a result of association analysis. One SNP circled in red has a high r^2^ and is found in the same region, indicating that the SNPs are highly correlated.

Table 1 shows details regarding the most significant SNP in Korean patients with gout. A meta-analysis was conducted using the 15 SNPs identified from the two cohorts. The rs1481012 SNP of the *ABCG2* gene (4:89039082_A/G) located on chromosome 4 satisfying the GWAS signature p threshold (*P*<5e^-8^) was discovered (*P* = 2.46e^-11^) (Figs 3 and 4).

**Table 1:** Significant SNPs related to gout established through meta-analysis.

CHR	POS	Gene	SNP	A1	A2	MAF	*P*
2	180988966	*CWC22*	rs17794144	A	G	0.06221	6.81E-05
3	64640212	*ADAMTS9*	rs59517147	T	G	0.2628	0.001627
3	77388130	*ROBO2*	rs146386352	T	G	0.05175	1.07E-05
4	9929575	*SLC2A9*	rs11936395	G	A	0.1355	0.004062
4	89039082	*ABCG2*	rs1481012	G	A	0.2723	2.46E-11
4	89064602	*ABCG2*	rs3109823	C	T	0.1607	0.000315
4	89170730	*PPM1K*	rs17013965	A	G	0.294	0.001446
6	11194628	*RP3-510L9*.*1*	rs3798728	A	T	0.3983	0.000356
6	35762473	*CLPS*	rs56205418	C	T	0.1601	4.34E-05
10	88879803	*FAM35A*	rs9421589	C	T	0.243	1.79E-05
10	125762202	*CHST15*	rs28674878	G	A	0.09973	0.002764
12	112930475	*PTPN11*	rs11066325	C	T	0.164	3.13E-06
13	38078153	*RP11-14O22*.*1*	rs9532070	T	C	0.4534	0.000986
19	6744762	*TRIP10*	rs339405	A	C	0.2231	6.89E-05
21	27296451	*APP*	rs200888518	G	C	0.1215	2.44E-05

*P*-value<5e^-8^

***CHR*** chromosome, ***POS*** position, ***SNP*** single nucleotide polymorphism, ***A1*** minor allele, ***A2*** major allele, ***MAF*** minor allele frequency, ***P*** = P-value

### Differences in PRS between the two groups

The PRS for the 15 lead SNPs was significantly higher for HEXA (*P* = 7.56E-63), and a higher PRS has been shown to affect gout (OR = 1.48, 95% CI = 1.41–1.55; *P* = 7.56e^-63^). However, for KARE, there was no significant differences between control group and patients with gout (S4 Table).

### Health-related lifestyle factors associated with gout

With respect to the number of exercise sessions per week, daily exercise was positively associated with the risk of gout (*P* = 0.036). For drinking behavior, past drinking and current drinking showed a 3.53-fold and 1.74-fold higher risk of gout, respectively (*P* = 5.23e^-10^, *P* = 2.56e^-05^, respectively). In particular, consuming more than 0.5 bottles of soju and beer increased the risk of gout by 0.30-fold and 0.38-fold, respectively (*P* = 0.000, *P* = 0.002, respectively).

Furthermore, current smoking was positively associated with risk of gout (*P* = 0.042). Subjects who had smoked for >41 years developed a 0.44-fold higher risk of gout than those who smoked for less than 5 years (*P* = 0.042). No significant differences in the risk of gout development were found with respect to eating habits (S5 Table).

### Interactions between 15 SNPs and health-related lifestyle factors

The interactions between SNPs and health-related lifestyle factors are presented in Table 2. The minor allele of rs3109823 located in *ABCG2* interacted with smoking status (OR = 0.73, 95% CI 0.54–0.98; *P* = 0.037). rs11936395 located in *SLC2A9* interacted with the average momentum of exercise per session (OR = 0.76, 95% CI 0.59–0.97; *P* = 0.033). The interaction between rs11066325 located in *PTPN11* and the number of exercise sessions per week, smoking status, drinking status, and amount of soju drink per session was significant. Duration of smoking was found to interact with rs9421589 located in *FAM35A* (OR = 1.18, 95% CI 1.01–1.39; *P* = 0.036).

**Table 2:** Interaction between 15 SNPs and health-related lifestyle factors.

CHR	BP	Gene	Minor allele	SNP	Health-related lifestyle factors	HEXA		CHR	BP	Gene	Minor allele	SNP	Health-related lifestyle factors	KARE	
						**OR (95% CI)**	***P***							**OR (95% CI)**	***P***
								**3**	77388130	*ROBO2*	T	rs146386352	smoking status	2.16 (1.42–3.28)	0.000
4	89064602	*ABCG2*	C	rs3109823	smoking status	0.73 (0.54–0.98)	0.037	4	89064602	*ABCG2*	C	rs3109823	smoking status	0.87 (0.61–1.25)	0.474
4	9929575	*SLC2A9*	G	rs11936395	the average momentum of exercise per session	0.76 (0.59–0.97)	0.033	4	9929575	*SLC2A9*	G	rs11936395	physical activity during the day (middle-intensity activity)	1.19 (1.00–1.41)	0.041
10	88879803	*FAM35A*	C	rs9421589	duration of smoking	1.18 (1.01–1.39)	0.036						smoking status	1.52 (1.11–2.07)	0.008
12	112930475	*PTPN11*	C	rs11066325	the number of exercise sessions per week	1.32 (1.02–1.70)	0.031	12	112930475	*PTPN11*	C	rs11066325	physical activity during the day (middle-intensity activity)	0.94 (0.78–1.13)	0.555
					smoking status	0.67 (0.51–0.89)	0.006						smoking status	0.77 (0.54–1.11)	0.171
					drinking status	0.79 (0.62–0.99)	0.048						drinking status	0.75 (0.53–1.06)	0.110
					the amount of soju drink per session	0.76 (0.58–0.99)	0.042	21	27296451	*APP*	G	rs200888518	Amount of drinking once of soju	1.415 (0.300–6.653)	0.660

*P*-value<0.05

***CHR*** chromosome, ***BP*** base pairs, ***SNP*** single nucleotide polymorphism, ***OR*** odds ratio, ***CI*** confidence interval, ***P*** P-value

covariants: age, sex, BMI

In KARE, the interaction between rs146386352 located in *ROBO2* and smoking status was significant (OR = 2.16, 95% CI 1.42–3.28; *P* = 0.000). In addition, rs11936395 located in *SLC2A9* was significantly associated with physical activity during the day (middle-intensity activity) as well as smoking status (*P* = 0.041, *P* = 0.008, respectively).

### Interaction between PRS and health-related lifestyle factors

Table 3 shows the analysis results of the interaction of PRS and health-related lifestyle factors. Among them, the duration of smoking did interact with PRS for the risk of gout (OR = 1.06, 95% CI = 1.00–1.11; *P* = 0.030).

**Table 3:** Interaction between PRS and health-related lifestyle factors.

Categories	HEXA		KARE	
	OR (95% CI)	*P*	OR (95% CI)	*P*
the number of exercise sessions per week	0.99 (0.94–1.05)	0.885	-	-
the average momentum of exercise per session	0.97 (0.92–1.04)	0.492	-	-
physical activity during the day (middle-intensity activity)	-	-	0.99 (0.95–1.03)	0.646
drinking status	1.03 (0.98–1.08)	0.221	1.00 (0.95–1.06)	0.793
the average frequency of soju consumed over the past year	1.02 (0.98–1.06)	0.268	1.14 (1.01–1.27)	0.023
the amount of soju drink per session	1.08 (0.96–1.22)	0.177	0.79 (0.54–1.12)	0.196
the amount of beer drink per session	0.97 (0.76–1.22)	0.795	0.81 (0.34–1.91)	0.634
smoking status	1.05 (1.00–1.10)	0.510	1.07 (1.00–1.16)	0.048
duration of smoking	1.06 (1.00–1.11)	0.030	1.18 (1.02–1.35)	0.019

*P*-value<0.05

***OR*** odds ratio, ***CI*** confidence interval, ***P*** P-value

Similarly, in KARE, the duration of smoking showed a significant association with gout risk (OR = 1.18 95% CI = 1.02–1.35; *P* = 0.019). Furthermore, the average number of soju drinks per year and smoking status were also significantly related with the risk of developing gout (*P* = 0.023, *P* = 0.048, respectively).

## Discussion

### SNPs related to gout

We identified 15 SNPs related to gout in the Korean population. rs1481012 was one of the 24 SNPs in *ABCG2* screened for their association with gout in the Chinese population [27], but its presence in Koreans was detected for the first time in this study. In previous studies, rs1481012 in ABCG2 has been associated with risk of hyperuricemia, gout, coronary artery disease (CAD), B-cell non-Hodgkin lymphoma (B-NHL), and chronic lymphocytic leukemia (CLL) [28–30]. Rs2728121 in *PKD2* and rs1481012 in *ABCG2* affect the etiology of diseases and are associated with elevated serum urate, hyperuricemia, and gout [5], which was in agreement with our findings that rs1481012 affects gout. We also identified rs3109823 in *ABCG2* and rs11936395 in *SLC2A9*. Several studies have established that *ABCG2* and *SLC2A9* are related to serum urate levels [6, 31]. Rs3109823 of *ABCG2* was also detected as one of the SNPs related to serum urate levels in Koreans [32]. Moreover, rs4529048 in *SLC2A9* affects serum urate levels. This is an iSNP located in the intron (DNA segment that does not contain genetic information or code for proteins) of *SLC2A9*, which also contains rs11936395 found in this study, and is associated with increased serum urate levels and risk of gout [33].

A study on Chinese patients revealed that the epistatic interactions between *PKD2* and *ABCG2* affect serum urate concentration and gout risk. This study confirmed that pairs of rs2728121 in *PKD2* and rs1481012 in *ABCG2* can impact the etiology of elevated serum urate, hyperuricemia, and gout [5], which supports the fact that rs1481012 discovered in this study influences gout. In addition, this study identified *APP*, *PTPN11*, *RP3-510L9*.*1*, *RP11-14O22*.*1*, *TRIP10*, *CHST15*, *CLPS*, *CWC22*, *PPM1*, *KADAMTS9*, *FAM35A*, and *ROBO2* as genes related to gout.

The amyloid precursor protein (*APP*) is located on chromosome 21 and is linked to gout as a genetic risk factor for Alzheimer’s disease, which can induce disease through interaction with the mutated *HPRT1* [34, 35]. Additionally, HPRT (hypoxanthine-guanine phosphoribosyltransferase) deficiency can be caused by a mutant *HPRT1* [36]. In absence of HPRT enzyme, hypoxanthine and guanine cannot be converted to nucleotides and are disintegrated in the form of uric acid, leading to an increase in serum urate concentration and abnormal accumulation of urate crystals in the kidneys and joints [37]. Ultimately, mutations in *HPRT1* can result in manifestation of disease through variations in the interaction between *HPRT1* and *APP* [38]. Protein tyrosine phosphatases (PTP) are known to regulate various processes such as cell growth, differentiation, division cycle, and tumor conversion [39]. We found in this study that *PTPN11* (protein tyrosine phosphatase non-receptor type 11), located on chromosome 12, interacts with three health-related lifestyle factors. In addition, mutations in *PTPN11* modulate serum HDL-C and are associated with hemostatic pathways such as platelet activation, aggregation, and sensitization [40, 41], as well as major genes associated with hypertension, ischemic heart disease, leukemia, and breast cancer [42]. Meanwhile, *PTPN11* encodes the Src homology 2 domain–containing protein phosphatase 2 (SHP-2), which mediates cell responses to various growth factors, hormones, and cytokines and is crucial for the migration and invasion of fibroblasts and tumor cells [42, 43]. SHP-2 enhances the survival and invasion of fibroblast-like synoviocytes (FLS) and the responsiveness to platelet-derived growth factor (PDGF) and tumor necrosis factor (TNF) in rheumatoid arthritis through the activation of focal adhesion kinase (FAK). It was shown that SHP-2 promotes the aggressiveness of FLS in rheumatoid arthritis [44]. These findings demonstrate that the *PTPN11* is associated with rheumatoid arthritis and that SHP-2 contributes to the development of rheumatoid arthritis [43]. *FAM35A* (family with sequence similarity 35 member A), located on chromosome 10, encodes proteins with 835 amino acids and is ubiquitous, including the kidneys and other organs such as thyroid, duodenum, and small intestine [45]. Recently, it was confirmed that rs7903456 of *FAM35A* was associated with hyperuricemia and gout in Japanese and Chinese populations [7]. Currently, there is no GWAS analysis on *FAM35A* in the Korean population. In this study, rs9421589 of *FAM35A* was identified as a significant lead SNP in the gout group. *ROBO2* on chromosome 3 encodes a transmembrane receptor for ligands and is essential for ureteric bud formation [46]. Mutations in this gene are associated with ureter reflux in the bladder, which is characterized by urine reflux from bladder to ureter or kidney [47]. Regarding this urinary reflux, mutations in the *ROBO2* are thought to be a potential genetic factor in patients with hyperuricemia and gout.

Minor allele frequency (MAF), the lower frequency of allelomorphic characters (alleles) in one SNP, is widely used in genetics because it distinguishes between common and rare variants in a population. MAF can vary in any population distribution as genetic variation and distribution vary with race [48]. Therefore, the prevalence of the disease may also vary with genetic variation and distribution can vary with race. According to a GWAS analysis in Han Chinese and Solomon Islanders, SNPs rs3733591, rs3733589, and rs1014290 in *SLC2A9* affect gout in Han Chinese but not in Solomon Islanders [49]. rs3733591 does not affect Polynesian or Caucasian gout patients [50]. Hence, the effect of SNPs in the same gene differ based on race, ethnicity, and region. Identification of SNPs through K-Chip provides a clearer understanding of the genetic factors of gout unique to Koreans. Further studies on other Korean population cohorts are needed to discover other genetic factors affecting gout in Koreans.

### Health-related lifestyle factors related to gout

The high purine content in beer may be partly responsible for increased risk of hyperuricemia/gout [51]. The major purine component in beer is guanine, and the increase in serum urate caused by purines contained in beer is sufficient to boost the effect of alcohol elevating serum urate levels [52, 53]. Ethanol from excessive consumption of soju/beer/distilled spirit causes hyperuricemia by decreasing urate excretion and increasing its production by enhancing ATP degradation to uric acid precursors [54]. Excessive ethanol consumption also causes a swift increase in alcohol metabolism (SIAM) thus inducing a hypermetabolic state with increased oxygen consumption in the liver cells primarily mediated by Kupffer cells, the resident hepatic macrophages [55]. Ethanol was reported to induce Kupffer cells to release prostaglandin E2 (PGE2), which stimulates glucose production from endogenous hepatic glycogen and increases oxygen uptake in hepatocytes leading to pericentral hypoxia [55]. Experimental hypoxia, correlated with preeclampsia, reportedly causes hyperuricemia [56]. In addition, previous studies have indicated that excessive consumption of soju (Korean distilled spirits) or other distilled spirits has been linked with an increased risk of gout, which supports our findings [52, 57]. Therefore, refraining from alcohol consumption can prevent gout.

Notably, we found that current smokers and those who have smoked for more than 41 years had a substantial positive correlation with gout-related SNPs. This differs from the findings of some studies on the association between gout and smoking. Previous research has found that the incidence of gout was 20–30% greater in nonsmokers than in smokers [58–60]. However, these studies did not consider genetic factors, limitedly considered the sex of the subjects, and the environmental factors considered were different for each study. Furthermore, several studies have noted the harmful effects of smoking and concluded that smoking was not recommended in patients with gout [58–60]. Therefore, the findings of this study are significant, suggesting that long-term smoking may increase the risk of gout-related genetic modification.

Purines are abundant in foods such as pork and beef, meat by-products (organ meat, blood, etc.), fish, shellfish, high-fructose fruits, and sugar or fructose-rich drinks [4]. Although several epidemiological studies have indicated that purine intake is associated with the incidence of gout [53, 61], purine intake and the number of regular meals per day were not associated with the gout-related SNPs in this study As purines are water-soluble, their concentration can be reduced by discarding the water during the cooking process. Moreover, by drinking more water, uric acid excretion can be increased by increasing urine discharge [62]. Hence, even if purine-rich foods are consumed, the degree of absorption may differ depending on the water intake pattern and cooking methods [63]. Therefore, to analyze the relationship between purine intake and gout more accurately, further studies focused on water intake and detailed cooking methods should be conducted.

In physical activity, the risk of gout was higher for those exercised for 61–120 min or for more than 120 min per session compared to those who exercised for less than 30 min. These results support those of a previous study in which people who exercised had a higher prevalence of gout than those who did not [64]. Furthermore, the prevalence of hyperuricemia was the highest when vigorous exercise was performed and decreased as exercise intensity decreased with moderate and mild exercise [65]. Several studies indicated that high-intensity exercise increases uridine and hypoxanthine levels in the blood, leading to an increase in serum urate concentration [65–67]. Therefore, it is recommended that patients with gout pay special attention to their exercise intensity and that further studies are needed on exercise interventions that consider the exercise intensity of patients with gout.

### Gout-related gene–lifestyle interactions and significance of PRS

The PRS of 15 SNPs related to gout were calculated, and their interactions with 8 health-related lifestyle factors were analyzed. The PRS associated with the duration of smoking, and if the smoking period was long, the genetic risk of gout increased, whereas if the smoking period was short, the genetic risk of gout was relatively low.

The PRS for the 15 gout-related lead SNPs was significantly higher in the gout group than in the control group (12.68±2.26 points in the gout group and 10.82±2.23 points in the control group in the HEXA cohort). Therefore, it is useful for predicting the genetic risk and its impact and for investigating gene–environment interactions. A single SNP that is associated with the outbreak of a disease has a low influence on diseases, whereas PRS assigns scores according to risk alleles for multiple SNPs and has a higher influence [68]. However, no gene–lifestyle interaction studies on gout have been reported in Korea. This study is significant as this study identified SNPs, for the first time, linking gout with health-related lifestyle factors.

## Conclusions

In conclusion, this study found, for the first time, that rs1481012 of *ABCG2* was associated with the risk of gout in Koreans. We also showed that genetic factors interact with health-related lifestyle factors such as lifestyle and have a significant impact on gout. In particular, the duration of smoking and amount of drinks consumed were shown to increase gout risk. These results suggest treatment and intervention require consideration of both genetic and lifestyle factors.

## Supporting information

S1 Fig Case/control selection process. (TIF)

S1 Table General and clinical characteristics. (DOCX)

S2 Table The significant 15 SNPs after discovery stage. (DOCX)

S3 Table Reproduced SNPs in KARE cohort after replication. (DOCX)

S4 Table Differences in PRS between the two groups. (DOCX)

S5 Table Health-related lifestyle factors associated with gout. (DOCX)
