# Supplementary figures and images for: Single-nucleotide polymorphisms link gout with health-related lifestyle factors in Korean cohorts
Source: PLoS One. 2023 Dec 7;18(12):e0295038. doi: 10.1371/journal.pone.0295038 (PMC10703335; doi:10.1371/journal.pone.0295038)

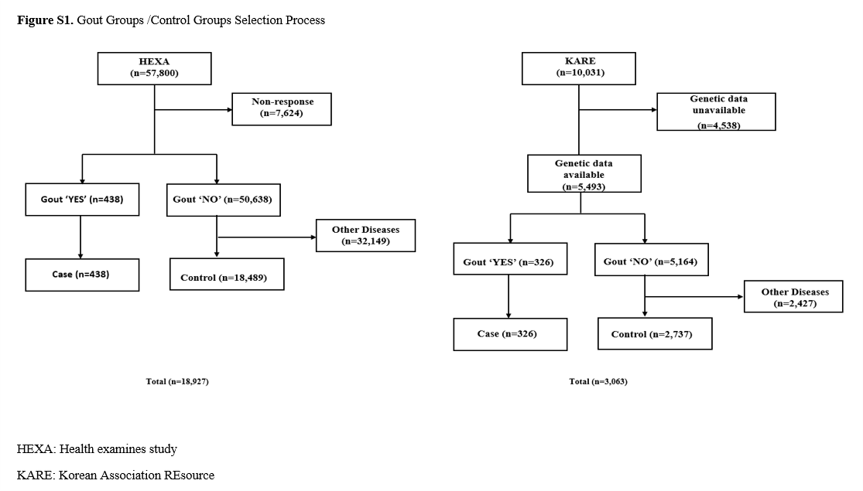

Supplement: S1 Fig — (TIF) [file pone.0295038.s001.tif]
