# Supplementary material for: Single-nucleotide polymorphisms link gout with health-related lifestyle factors in Korean cohorts
Source: PLoS One. 2023 Dec 7;18(12):e0295038. doi: 10.1371/journal.pone.0295038 (PMC10703335; doi:10.1371/journal.pone.0295038)
Supplement: S1 Table — (DOCX) [file pone.0295038.s002.docx]

| **Table S1.** General and Clinical Characteristics of Participants | | | | | |
| --- | --- | --- | --- | --- | --- |
| Variables | Categories | Gout | | t/*x*^2^ | *P* |
|  |  | Gout Groups  (n=438) | Control Groups (n=18,489) |  |  |
|  |  | n(%) or M±SD | n(%) or M±SD |  |  |
| Gender | Male | 369(84.2) | 5,707(30.9) | 559.32 | <.001 |
|  | Female | 69(15.8) | 12,782(69.1) |  |  |
| Age(year) |  | 56.93±7.45 | 50.53±7.39 | -17.90 | <.001 |
| Marital status | Unmarried | 5(1.1) | 527(2.9) | 4.84 | .089 |
|  | Married | 403(92.4) | 16,837(91.4) |  |  |
|  | Others | 28(6.4) | 1,055(5.7) |  |  |
| Occupation | Yes | 281(64.9) | 10,740(58.9) | 6.37 | .012 |
|  | No | 152(35.1) | 7,507(41.1) |  |  |
| Education | ＜Elementary school | 8(1.8) | 225(1.2) | 22.24 | <.001 |
|  | Elementary school  (<Middle school) | 43(9.9) | 1,505(8.2) |  |  |
|  | Middle school  (<High school) | 55(12.7) | 2,465(13.5) |  |  |
|  | High school <College) | 161(37.1) | 8,631(47.1) |  |  |
|  | ≥Bachelor | 167(38.5) | 5,496(30.0) |  |  |
| Household  monthly income  (10,000 won) | ＜100 | 46(10.8) | 1,203(6.8) | 16.22 | .003 |
|  | ＜200 | 81(19.0) | 3,118(17.6) |  |  |
|  | ＜400 | 168(39.4) | 8,360(47.1) |  |  |
|  | ＜600 | 88(20.7) | 3,411(19.2) |  |  |
|  | ≥600 | 43(10.1) | 1,646(9.3) |  |  |
| Age of gout  diagnosis(year) | 50.31±9.02 | - | - | - |  |
| Height(cm) | 161.10±7.96 | 160.70±7.89 | -.22 | .826 |  |
| Weight(kg) | 61.90±10.09 | 61.79±9.84 | -.22 | .826 |  |
| BMI(kg/m2) | 23.76±2.79 | 23.86±2.89 | .70 | .482 |  |
| SBP(mmHg) |  | 122.69±14.27 | 122.09±14.87 | -.83 | .405 |
| DBP(mmHg) |  | 76.11±9.55 | 75.49±9.69 | -1.32 | .185 |
| Pulse(/1min) |  | 68.44±8.81 | 69.10±8.90 | 1.53 | .126 |
| FBS(mg/dl) |  | 98.39±19.89 | 91.12±13.84 | -7.52 | <.001 |
| BUN(mg/dl) |  | 16.19±6.91 | 13.85±3.65 | -7.03 | <.001 |
| Cr(mg/dl) |  | 1.05±0.47 | 0.77±0.18 | -12.25 | <.001 |
| UA(mg/dl) |  | 6.67±1.84 | 4.51±1.18 | -24.46 | <.001 |
| TC(mg/dl) |  | 191.60±36.4 | 197.59±33.90 | 3.40 | .001 |
| HDL(mg/dl) |  | 47.47±11.35 | 55.38±13.47 | 14.33 | <.001 |
| LDL(mg/dl) |  | 111.55±32.92 | 119.57±30.24 | -9.48 | <.001 |
| TG(mg/dl) |  | 173.03±124.32 | 116.32±80.39 | 4.92 | <.001 |
| AST(IU/L) |  | 26.13±11.44 | 22.50±10.74 | -6.48 | <.001 |
| ALT(IU/L) |  | 26.74±15.46 | 20.54±15.69 | -8.18 | <.001 |
| TB(mg/dl) |  | 0.77±0.28 | 0.73±0.30 | -1.95 | .051 |
| hs-CRP(mg/dl) |  | 0.23±0.52 | 0.11±0.29 | -4.10 | <.001 |

***M*** Mean, ***SD*** Standard Deviation, ***P*** P-value, ***HTN*** hypertension, ***DM*** diabetes mellitus, ***BMI*** body mass index, ***SBP*** systolic blood pressure, ***DBP*** diastolic blood pressure, ***FBS*** fasting blood sugar, ***BUN*** blood urea nitrogen, ***Cr*** creatinine, ***UA*** uric acid, ***TC*** total cholesterol, ***HDL*** high density lipoprotein, ***LDL*** low density lipoprotein, ***TG*** triglycerides, ***AST*** aspartate transaminase, ***ALT*** Alanine Transaminase, ***TB*** Total Bilirubin, ***hs-CRP*** high sensitivity C-reactive protein.
