# Supplementary material for: Single-nucleotide polymorphisms link gout with health-related lifestyle factors in Korean cohorts
Source: PLoS One. 2023 Dec 7;18(12):e0295038. doi: 10.1371/journal.pone.0295038 (PMC10703335; doi:10.1371/journal.pone.0295038)
Supplement: S2 Table — (DOCX) [file pone.0295038.s003.docx]

**Table S2.** The Significant 15 SNPs after Discovery Stage

| CHR | POS | Gene | SNP | Minor allele | MAF | OR (95%CI) | *P* |
| --- | --- | --- | --- | --- | --- | --- | --- |
| 2 | 180988966 | *CWC22* | rs17794144 | A | 0.1005 | 1.69(1.34-2.13) | 7.90E-06 |
| 3 | 64640212 | *ADAMTS9* | rs59517147 | T | 0.1929 | 0.67(0.56-0.80) | 8.00E-06 |
| 3 | 77388130 | *ROBO2* | rs146386352 | T | 0.0799 | 1.80(1.38-2.34) | 9.76E-06 |
| 4 | 9929575 | *SLC2A9* | rs11936395 | G | 0.1941 | 1.54(1.29-1.83) | 1.37E-06 |
| 4 | 89039082 | *ABCG2* | rs1481012 | G | 0.3870 | 1.76(1.52-2.03) | 1.12E-14 |
| 4 | 89064602 | *ABCG2* | rs3109823 | C | 0.1073 | 0.60(0.48-0.75) | 6.02E-06 |
| 4 | 89170730 | *PPM1K* | rs17013965 | A | 0.2317 | 0.68(0.58-0.81) | 7.98E-06 |
| 6 | 11194628 | *RP3-510L9.1* | rs3798728 | A | 0.4749 | 1.39(1.21-1.59) | 2.80E-06 |
| 6 | 35762473 | *CLPS* | rs56205418 | C | 0.2169 | 1.47(1.24-1.73) | 6.98E-06 |
| 10 | 88879803 | *FAM35A* | rs9421589 | C | 0.3265 | 1.49(1.28-1.73) | 9.42E-08 |
| 10 | 125762202 | *CHST15* | rs28674878 | G | 0.1438 | 1.57(1.29-1.91) | 6.72E-06 |
| 12 | 112930475 | *PTPN11* | rs11066325 | C | 0.1221 | 0.59(0.48-0.74) | 2.00E-06 |
| 13 | 38078153 | *RP11-14O22.1* | rs9532070 | T | 0.3824 | 0.72(0.62-0.83) | 5.71E-06 |
| 19 | 6744762 | *TRIP10* | rs339405 | A | 0.2831 | 1.43(1.22-1.67) | 6.50E-06 |
| 21 | 27296451 | *APP* | rs200888518 | G | 0.1678 | 1.57(1.30-1.89) | 1.84E-06 |

*P<*1e^-5^

***CHR*** chromosome, ***POS*** position, ***SNP*** single nucleotide polymolphism, ***MAF*** minor allele frequency, ***OR*** odds ratio, ***CI*** confidence interval, ***P*** P-value
