# Supplementary material for: Single-nucleotide polymorphisms link gout with health-related lifestyle factors in Korean cohorts
Source: PLoS One. 2023 Dec 7;18(12):e0295038. doi: 10.1371/journal.pone.0295038 (PMC10703335; doi:10.1371/journal.pone.0295038)
Supplement: S3 Table — (DOCX) [file pone.0295038.s004.docx]

**S3 Table.** Reproduced SNPs in KARE Cohort after Replication Stage

| CHR | POS | Gene | SNP | Minor allele | MAF | OR (95%CI) | *P* |
| --- | --- | --- | --- | --- | --- | --- | --- |
| 2 | 180988966 | *CWC22* | rs17794144 | A | 0.0720 | 1.10(0.79-1.52) | 0.561 |
| 3 | 64640212 | *ADAMTS9* | rs59517147 | T | 0.2745 | 1.01(0.84-1.23) | 0.841 |
| 3 | 77388130 | *ROBO2* | rs146386352 | T | 0.0567 | 1.29(0.89-1.88) | 0.170 |
| 4 | 9929575 | *SLC2A9* | rs11936395 | G | 0.1104 | 0.76(0.58-0.98) | 0.039 |
| 4 | 89039082 | *ABCG2* | rs1481012 | G | 0.2929 | 1.08(0.90-1.31) | 0.373 |
| 4 | 89064602 | *ABCG2* | rs3109823 | C | 0.1518 | 0.94(0.75-1.20) | 0.663 |
| 4 | 89170730 | *PPM1K* | rs17013965 | A | 0.2975 | 1.02(0.85-1.23) | 0.802 |
| 6 | 11194628 | *RP3-510L9.1* | rs3798728 | A | 0.4034 | 0.98(0.82-1.17) | 0.863 |
| 6 | 35762473 | *CLPS* | rs56205418 | C | 0.1718 | 1.09(0.87-1.37) | 0.416 |
| 10 | 88879803 | *FAM35A* | rs9421589 | C | 0.2331 | 0.99(0.81-1.21) | 0.982 |
| 10 | 125762202 | *CHST15* | rs28674878 | G | 0.0920 | 0.82(0.61-1.10) | 0.199 |
| 12 | 112930475 | *PTPN11* | rs11066325 | C | 0.1426 | 0.81(0.64-1.03) | 0.096 |
| 13 | 38078153 | *RP11-14O22.1* | rs9532070 | T | 0.4586 | 1.02(0.86-1.21) | 0.755 |
| 19 | 6744762 | *TRIP10* | rs339405 | A | 0.2423 | 1.07(0.88-1.30) | 0.473 |
| 21 | 27296451 | *APP* | rs200888518 | G | 0.1304 | 1.08(0.84-1.40) | 0.528 |

*P* <0.05/15

***CHR*** chromosome, ***POS*** position, ***SNP*** single nucleotide polymolphism, ***MAF*** minor allele frequency, ***OR*** odds ratio, ***CI*** confidence interval, ***P*** P-value
