# Supplementary material for: Single-nucleotide polymorphisms link gout with health-related lifestyle factors in Korean cohorts
Source: PLoS One. 2023 Dec 7;18(12):e0295038. doi: 10.1371/journal.pone.0295038 (PMC10703335; doi:10.1371/journal.pone.0295038)
Supplement: S4 Table — (DOCX) [file pone.0295038.s005.docx]

**S4 Table.** Differences in the PRS between HEXA and KARE Cohorts

| **Variables** | **HEXA** | | | | | **KARE** | | | | |
| --- | --- | --- | --- | --- | --- | --- | --- | --- | --- | --- |
|  | **Total**  **(n=18927)** | **Gout**  **(n=438)** | **Control**  **(n=18489)** | **OR (95% CI)** | **P** | **Total**  **(n=6063)** | **Gout**  **(n=326)** | **Control**  **(n=2737)** | **OR (95% CI)** | ***P*** |
|  | **M±SD** | **M±SD** | **M±SD** |  |  | **M±SD** | **M±SD** | **M±SD** |  |  |
| PRS | 10.86±2.25 | 12.68±2.26 | 10.82±2.23 | 1.481 (1.41-1.55) | 7.56E-63 | 10.89±2.25 | 10.96±2.18 | 10.89±2.21 | 1.014 (0.963-1.068) | .598 |

*P*-value<0.05

***HEXA*** Health Examinees study, ***KARE*** Korean Association Resource, ***PRS*** polygenic risk score, ***M*** Mean, ***SD*** Standard Deviation,

***OR*** odds ratio, ***CI*** confidence interval, ***P*** P-value
