# Supplementary material for: Single-nucleotide polymorphisms link gout with health-related lifestyle factors in Korean cohorts
Source: PLoS One. 2023 Dec 7;18(12):e0295038. doi: 10.1371/journal.pone.0295038 (PMC10703335; doi:10.1371/journal.pone.0295038)
Supplement: S5 Table — (DOCX) [file pone.0295038.s006.docx]

**S5 Table.** Health-related Lifestyle Factors Associated with Gout

| Variables | Categories | β | SE | OR (95% CI) | *P* |
| --- | --- | --- | --- | --- | --- |
| **Eating habits** | | | | | |
| The number of regular meals per day | 1 time |  |  | ref. | 0.561 |
|  | 2 time | -0.257 | 0.757 | 0.77(0.18-3.41) | 0.734 |
|  | 3 time | -0.421 | 0.749 | 0.65(0.15-2.85) | 0.574 |
|  | 4 time or more | -0.687 | 0.875 | 0.50(0.09-2.80) | 0.433 |
|  | irregular | 0.416 | 0.983 | 1.51(0.24-9.53) | 0.658 |
| The average frequency of pork belly intake over the past year | Hardly eat |  |  | ref. | 0.736 |
|  | 1-3 times a month | -0.117 | 0.131 | 0.89(0.69-1.15) | 0.374 |
|  | 1-4 times a week | 0.038 | 0.154 | 1.03(0.77-1.41) | 0.803 |
|  | 5-6 times a week | 0.248 | 1.038 | 1.28(0.17-9.79) | 0.811 |
|  | 1-3 times a day | -17.250 | 5446.016 | - | 0.953 |
| The average frequency of beef intake over the past year | Hardly eat |  |  | ref. | 0.854 |
|  | 1-3 times a month | -0.034 | 0.106 | 0.96(0.79-1.19) | 0.751 |
|  | 1-4 times a week | -0.192 | 0.197 | 0.82(0.56-1.21) | 0.330 |
|  | 5-6 times a week | 0.630 | 1.056 | 1.87(0.24-14.88) | 0.551 |
|  | 1-3 times a day | -17.068 | 6090.494 | - | 0.998 |
| The average frequency of intestines intake over the past year | Hardly eat |  |  | ref. | 0.936 |
|  | 1-3 times a month | -0.067 | 0.018 | 0.93(0.76-1.15) | 0.531 |
|  | 1-4 times a week | 0.163 | 0.2861.177 | 1.17(0.67-2.06) | 0.568 |
|  | 5-6 times a week | -17.585 | 14384.894 | - | 0.982 |
|  | 1-3 times a day | -16.512 | 9105.151 | - | 0.974 |
| The average frequency of seafood(shellfish/whelk) intake over the past year | Hardly eat |  |  | ref. | 0.734 |
|  | 1-3 times a month | 0.071 | 0.108 | 1.07(0.87-1.33) | 0.511 |
|  | 1-4 times a week | -0.004 | 0.161 | 0.99(0.73-1.37) | 0.981 |
|  | 5-6 times a week | 0.309 | 0.735 | 1.36(0.32-5.76) | 0.674 |
|  | 1-3 times a day | 0.658 | 0.539 | 1.93(0.67-5.55) | 0.223 |
| The average frequency of shrimp intake over the past year | Hardly eat |  |  | ref. |  |
|  | 1-3 times a month | -0.109 | 0.104 | 0.89(0.73-1.10) | 0.296 |
|  | 1-4 times a week | 0.305 | 0.217 | 1.35(0.89-2.07) | 0.160 |
|  | 5-6 times a week | 0.176 | 1.047 | 1.19(0.15-9.27) | 0.867 |
|  | 1-3 times a day | 0.805 | 0.760 | 2.23(0.50-9.92) | 0.289 |
| The average frequency of coffee intake over the past year | Hardly eat |  |  | ref. |  |
|  | 1-3 times a month | 0.223 | 0.244 | 1.24(0.77-2.02) | 0.362 |
|  | 1-4 times a week | -0.028 | 0.201 | 0.97(0.66-1.44) | 0.887 |
|  | 5-6 times a week | -0.015 | 0.314 | 0.98(0.53-1.82) | 0.961 |
|  | 1-3 times a day | 0.085 | 0.141 | 1.08(0.83-1.44) | 0.546 |
| The average frequency of soft drink intake over the past year | Hardly eat |  |  | ref. |  |
|  | 1-3 times a month | 0.032 | 0.131 | 1.03(0.80-1.33) | 0.810 |
|  | 1-4 times a week | 0.050 | 0.198 | 1.05(0.71-1.55) | 0.800 |
|  | 5-6 times a week | -17.498 | 5411.566 | - | 0.997 |
|  | 1-3 times a day | -17.174 | 3578.875 | - | 0.996 |
| **Physical activity** | | | | | |
| Exercise status | No |  |  | ref. |  |
|  | Yes | 0.190 | 0.102 | 1.20(0.99-1.48) | 0.064 |
| The number of exercises per week | 1-4 times a week |  |  | ref. |  |
|  | 5-6 times a week | 0.192 | 0.175 | 1.21(0.85.-1.71) | 0.273 |
|  | Daily | 0.437 | 0.208 | 1.54(1.02-2.32) | 0.036 |
| The average momentum of exercise once | ≤ 30 min |  |  | ref. |  |
|  | 31 min-60 min | 0.505 | 0.312 | 1.65(0.89-3.05) | .105 |
|  | 61 min-120 min | 1.042 | 0.246 | 2.83(1.75-4.59) | .000 |
|  | ≥121 min | 0.682 | 0.256 | 1.97(1.19-3.26) | .008 |
| **Drinking behavior** | | | | | |
| Drinking status | non-drinking |  |  | ref. |  |
|  | past drinking | 1.259 | 0.203 | 3.52(2.37-5.24) | 5.23E-10 |
|  | current drink | 0.556 | 0.132 | 1.74(1.35-2.26) | 2.56E-5 |
| Duration of drinking | 1-5 years |  |  | ref. |  |
|  | 6-10 years | 0.285 | 0.492 | 1.33(0.51-3.49) | 0.562 |
|  | 11-20 years | 0.335 | 0.446 | 1.39(0.58-3.35) | 0.453 |
|  | 21-30 years | 0.293 | 0.435 | 1.34(0.57-3.14) | 0.500 |
|  | 31-40 years | 0.367 | 0.438 | 1.44(0.61-3.40) | 0.402 |
|  | over 41 years | 0.546 | 0.460 | 1.72(0.70-4.25) | 0.235 |
| The average frequency of soju consumed over the past year | don't drink |  |  | ref. |  |
|  | 1-3 times a month | 0.233 | 0.150 | 1,26(0.94-1.69) | 0.121 |
|  | 1-3 times a week | 0.455 | 0.124 | 1.57(1.23-2.01) | 2.58E-04 |
|  | 4-6 times a week | 0.615 | 0.213 | 1.84(1.22-2.81) | 0.004 |
|  | ≥ 1-2 times daily | 0.230 | 0.318 | 1.25(0.67-2.35) | 0.470 |
| The amount of drinking once of soju | ≤ 0.5 bottle |  |  | ref. |  |
|  | 0.5 ＜bottles ≤ 1 | -1.175 | 0.212 | 0.30(0.20.-0.46) | 0.000 |
|  | 1＜bottles ＜2 | -0.752 | 0.218 | 0.47(0.30-0.72) | 0.001 |
|  | ≥ 2 bottles | -0.408 | 0.196 | 0.66(0.45-0.97) | 0.038 |
| The average frequency of beer consumed over the past year | don't drink |  |  | ref. |  |
|  | 1-3 times a month | -0.094 | 0.160 | 0.91(0.67-1.24) | 0.554 |
|  | 1-3 times a week | 0.088 | 0.144 | 1.09(0.82-1.45) | 0.543 |
|  | 4-6 times a week | -0.234 | 0.521 | 0.79(0.29-2.19) | 0.653 |
|  | ≥ 1-2 times daily | 0.381 | 0.535 | 1.46(0.51-4.18) | 0.477 |
| The amount of drinking once of beer | ≤ 0.5 bottle |  |  | ref. |  |
|  | 0.5 ＜bottles ≤ 1 | -0.954 | 0.315 | 0.38(0.20-0.71) | 0.002 |
|  | 1＜bottles ＜2 | -0.668 | 0.222 | 0.51(0.33-0.79) | 0.003 |
|  | ≥ 2 bottles | -1.070 | 0.306 | 0.34(0.18-0.62) | 0.000 |
| **Smoking behavior** | | | | | |
| Smoking status | non-smoking |  |  | ref. |  |
|  | past smoking | 1.076 | 0.276 | 1.01(0.75-1.35) | 0.917 |
|  | current smoking | 0.017 | 0.297 | 1.31(1.01-1.72) | 0.042 |
| Duration of smoking | 1-5 years |  |  | ref. |  |
|  | 6-10 years | 0.068 | 0.336 | 1.07(0.55-2.06) | 0.840 |
|  | 11-20 years | -0.354 | 0.295 | 0.70(0.39-1.25) | 0.229 |
|  | 21-30 years | -0.142 | 0.278 | 0.86(0.50-1.49) | 0.609 |
|  | 31-40 years | -0.253 | 0.291 | 0.77(0.44-1.37) | 0.385 |
|  | over 41 years | -0.804 | 0.396 | 0.44(0.20-0.97) | 0.042 |
| The number of cigarettes smoked per day | 1-10 cigarettes |  |  | ref. | 0.312 |
|  | 11~20 cigarettes | 0.192 | 0.145 | 1.21(0.91-1.61) | 0.184 |
|  | 21~30 cigarettes | 0.451 | 0.253 | 1.57(0.96-2.58) | 0.074 |
|  | 31~40 cigarettes | 0.363 | 0.305 | 1.43(0.79-2.61) | 0.233 |
|  | more than 41 cigarettes | 0.551 | 0.548 | 1.73(0.59-5.07) | 0.314 |

*P*-value<0.05

***Β*** beta, ***SE*** standard error, ***OR*** odds ratio, ***CI*** confidence interval, ***P*** P-value

covariant : age, sex, BMI
